# Are outpatient costs for hypertension and diabetes care affordable? Evidence from Western Kenya

**DOI:** 10.4102/phcfm.v15i1.3889

**Published:** 2023-09-29

**Authors:** Mwaleso Kishindo, Jemima Kamano, Ann Mwangi, Thomas Andale, Grace W. Mwaura, Obed Limo, Kenneth Too, Richard Mugo, Ephantus Maree, Wilson Aruasa

**Affiliations:** 1 Academic Model Providing Access to Healthcare, Eldoret, Kenya; 2 School of Medicine, Moi University, Eldoret, Kenya; 3 Division of Non-Communicable Disease, Ministry of Health, Nairobi, Kenya; 4 Department of Mathematics, Physics, and Computing, Moi University, Eldoret, Kenya; 5 Moi Teaching and Referral Hospital, Eldoret, Kenya

**Keywords:** out-patient costs, non-communicable diseases, catastrophic healthcare expenditure, primary healthcare, comorbidity

## Abstract

**Background:**

Diabetes and hypertension pose a significant socio-economic burden in developing countries such as Kenya, where financial risk-protection mechanisms remain inadequate. This proves to be a great barrier towards achieving universal health care in such settings unless mechanisms are put in place to ensure greater access and affordability to non-communicable disease (NCD) management services.

**Aim:**

This article aims to examine outpatient management services costs for patients with diabetes and hypertension attending public primary healthcare facilities.

**Setting:**

The study was conducted in Busia and Trans-Nzoia counties in Western Kenya in facilities supported by the PIC4C project, between August 2020 and December 2020.

**Methods:**

This cross-sectional survey included 719 adult participants. Structured interviewer-administered questionnaires were used to collect information on healthcare-seeking behaviour and associated costs. The annual direct and indirect costs borne by patients were computed by disease type and level of healthcare facility visited.

**Results:**

Patients with both diabetes and hypertension incurred higher annual costs (KES 13 149) compared to those with either diabetes (KES 8408) or hypertension (KES 7458). Patients attending dispensaries and other public healthcare facilities incurred less direct costs compared to those who visited private clinics. Furthermore, a higher proportionate catastrophic healthcare expenditure of 41.83% was noted among uninsured patients.

**Conclusion:**

Despite this study being conducted in facilities that had an ongoing NCDs care project that increased access to subsidised medication, we still reported a substantially high cost of managing diabetes and hypertension among patients attending primary healthcare facilities in Western Kenya, with a greater burden among those with comorbidities.

**Contribution:**

Evidenced by the results that there is enormous financial burden borne by patients with chronic diseases such as hypertension and diabetes; we recommend that universal healthcare coverage that offers comprehensive care for NCDs be urgently rolled out alongside strengthening of lower-level public healthcare systems.

## Introduction

Non-communicable diseases (NCDs) are increasingly imposing a greater disease and economic burden globally.^1^ For instance, evidence from the 2019 Global Burden of Disease study indicated that nine out of the top 10 drivers of increasing disability-adjusted life years (DALYs) were NCDs.^2^ With the increasing costs associated with accessing healthcare^3^ especially in developing countries,^4^ NCDs would further burden developing countries and derail the move towards universal health coverage (UHC).

Universal health coverage is a primary target of the Sustainable Development Goal 3, which compels countries to streamline their health systems to ensure that everyone has access to good quality healthcare services that they need without experiencing any financial hardship as a result.^5,6,7^ Central to UHC is ensuring that all citizens are protected from financial ruin resulting from accessing healthcare.^8^ However, financial risk protection mechanisms remain hardly adopted in most developing countries. For example, only four countries in sub-Saharan Africa have health insurance coverage greater than 20%.^9^ Consequently, financing of healthcare has largely depended on out-of-pocket payments (OOP) and donors. For instance, 39.6% and 23.4% of Kenya’s total health expenditure in 2016 was composed OOP and donor funding, respectively.^10^

The higher dependence on OOP as a financing mechanism results in a higher incidence of catastrophic health expenditure (CHE) (defined as OOP health payments exceeding a certain threshold of household’s capacity to pay or non-subsistence spending)^11,12^ and impoverishments,^13^ especially among patients with NCD. Evidence from a study in Kenya indicated that nearly three in five patients with hypertension experienced CHE as a result of direct healthcare costs.^14^ In another study among patients with diabetes in Kenya, the incidence of CHE was 75.4%.^15^

Despite Kenya’s commitment to achieve UHC by 2022^16^ and the prioritisation to deliver NCD services through primary health care (PHC) facilities (level 1–3) – dispensaries and health centres, treatment for NCDs has traditionally been available only in level 4–6 facilities – secondary (county) and tertiary (national) referral hospitals. As a result, very little effort has been put towards community prevention activities, early detection and treatment or improved continuity of care for NCDs; yet these elements are fundamental for successfully addressing the threat of NCDs. It remains unclear how much it costs patients with comorbidities to receive services from these facilities. Although some patient cost of illness studies exists in Kenya, they have focused on single diseases such as diabetes^15^ and hypertension^14^ separately, yet comorbidities such as hypertension and diabetes often exist. Furthermore, these studies have generally looked at aggregate costs of the diseases at secondary and tertiary facilities.

Against this backdrop, this study aims to examine the cost of outpatient services that patients with two NCDs, diabetes and hypertension, incur to access services at PHC facilities in two Western Kenya counties. Diabetes and hypertension are important modifiable risk factors for cardiovascular disease (CVD), which accounts for over 29% of global deaths.^17^ They also have shared pathways of complications and clinical approaches.^18,19^ This presents an opportunity for a unified model of care at the PHC level to alleviate a considerable cause of mortality and disability.^20^ The focus is on outpatient visits as they form the largest share of interaction between patients with NCDs and the health care system and because there is an emphasis on providing these services at PHC facilities that are often closer to patients than secondary facilities. This study aims to generate evidence essential for adequately understanding the cost of delivering these services at PHC facilities. It is our hope that these data will facilitate policy making efforts aimed at increasing access to care of these two conditions at the PHC level in line with the WHO Package of Essential Non-communicable Disease Interventions (WHO PEN) for Primary Care in low-resource settings.^21^

## Research methods and design

### Study design

This was a cross-sectional survey.

### Setting

The study was nested within a pilot implementation program that sought to integrate promotive, curative care for diabetes, hypertension, breast and cervical cancer at the PHC level (Primary Health Integrated Care for Chronic Conditions, PIC4C) within the Academic Model providing Access to Healthcare (AMPATH) in western Kenya.^22,23,24,25^ The PIC4C program was piloted in Busia and Trans-Nzoia counties for their unique peculiarity of high disease burden,^26^ and a strong commitment offered by the two county governments towards strengthening health systems management of NCDs via increased county budget allocation^27^ The two counties are part of AMPATH catchment area having some health facilities offering chronic disease management program through specialised clinics for diabetes and hypertension management, 2 days a week.

### Study population

We included patients with diabetes, hypertension and both hypertension and diabetes who had actively sought care within the last 6 months in the selected health facilities. All consenting adults who were 18 years and above were included in this study.

### Sample size estimation

The targeted sample size was 768 (384 per county) based on a conservative assumption of population disease proportion of 50%, a confidence interval (CI) of 95% and a precision of 0.05.^28^ The formula used to estimate the required sample was:^29^

$$n={\left(\text{Z}\right)}^{2}\left(P\left(1-P\right)\right)/d^{2}$$ [Eqn 1]

### Sampling procedure

A stratified purposive sampling strategy was used to select a total of 22 health facilities in the two counties based on the levels, workload and spatial distribution: eight sub-county hospitals, four health centres, eight dispensaries and two private clinics.^29^

Patients were recruited on clinic days within the selected health facilities by research assistants through simple random sampling of the eligible patients. The interviews were conducted while the patients were awaiting their consultations. No inducement or incentive was offered for participation.

### Data collection

Data were collected using a structured questionnaire in English or Kiswahili language where applicable using mobile tablets. Respondents were asked to recall and report on their health service utilisation and costs incurred for the different cost types while seeking outpatient care in the selected facilities (Table 1).

**TABLE 1:** Description of cost category estimates.

Cost type	Cost component	Cost estimation approach	Recall period
Direct	Direct medical	**Medicines:** Computed as a sum of costs of medicines prescribed to patients.	1 month
		**Out-patient costs:** These were the sum of consultation, investigation and any other costs other than medical costs that patients received.	4 months
		**Total medical costs:** This was the sum of medicines and out-patient costs.	-
	Direct non-medical	**Transport:** These were costs incurred to and from the facility.	4 months
	Total direct costs	This was the sum of direct medical and non-medical costs.	-
Indirect	Productivity losses because of seeking health care	This was foregone income by patients because of care-seeking.	4 months
	Other productivity losses	Was computed as a sum of costs incurred where a caregiver was hired.	4 months
	Total indirect costs	This was the sum of productivity losses.	-
Direct and Indirect	The overall cost for NCD management	This was the sum of direct and indirect costs.	-

### Data analysis

Costs for each cost component described in Table 1 were summed up and annuitised following their respective recall period. For instance, medicine costs were annuitised by multiplying the summed-up cost by 12 as the recall period was monthly. Further, costs were broadly categorised into either direct or indirect costs. Direct costs comprised outright OOP costs patient paid to access care such as the medicines, consultation and investigation costs (cumulatively here referred to as direct medical costs) and the transport cost (hereafter referred as direct non-medical cost) incurred to and from the health facility. On the other hand, indirect costs are related to the productivity losses associated with the patient’s foregone income because of seeking care and the associated caregiver costs (in cases where caregivers were engaged). Indirect costs were estimated by calculating the total number of hours a patient spent while seeking care. Productivity losses for the unemployed were estimated using the median income.^30^ A workday was defined to have 8 h while a month was assumed to have 22 working days. Caregiver costs were annuitised by adding up the costs paid by the patient to engage the caregivers.

Wealth quintiles were calculated using the principal component analysis.^31^ Income was estimated by asking patients about their income categories or brackets and then assigning the mean in each of the categories. The median income was assigned to patients who did not know or report their incomes. To examine the incidence of CHE, the total annual direct costs incurred by the patient was compared against the annual household income. Catastrophic health expenditure was defined as utilisation of more than 10% of total annual household income on direct medical costs.^11,32,33^ In this study, we restricted CHE analysis to patients who were uninsured by the national insurance scheme (NHIF). Those with private insurance were included as there may be a chance for co-payment in public facilities.

Two approaches were employed to examine the inequalities in the incidence of CHE across the different disease categories. Firstly, concentration curves^11^ for the CHE were constructed by specific disease condition and overall. A concentration curve is a plot of the cumulative percentage of a variable of interest (CHE) (y-axis) against the cumulative proportion of the population, ranked by wealth status, from the poorest to the richest (x-axis). The concentration curve is a 45-degree line (line of equality) when every individual, irrespective of their socio-economic status, receives the same value of the variable of interest, but a curve that lies below (above) the line of equality indicates that the variable of interest is pro-rich (pro-poor).^11^ Secondly, the Wagstaff concentration index was generated, which is mathematically defined as twice the area between the concentration curve and the line of equality. A zero value indicates equality, whereas a negative (positive) value indicates that the variable of interest is more concentrated among the poor (rich).^11^

Each questionnaire was automatically uploaded to a REDCap database where a data manager inspected to identify any issues or missing data and eliminated data errors. Data completeness was ascertained by examining data consistency and plausible ranges. Data were analysed using STATA 16.1.^34^ Frequency counts and percentages were generated to present the distribution of patient characteristics across selected demographic and socio-economic factors. Costs were calculated and presented as means and medians with their 95% CI and interquartile range (IQR). The costs were converted from Kenya Equivalent Shilling (KES) into 2020 United States Dollars ($) using the following exchange rate: $ 1.00 = KES 108.315.^35^

### Ethical considerations

The study received ethics approval from the Moi Teaching and Referral Hospital or Moi University School of Medicine Institutional Research and Ethics Committee (IREC) (approval number 0002090). Furthermore, permission to conduct the study was also sought from National Commission for Science Technology and Innovation (NACOSTI) (approval number NACOSTI/P/18/74238/24329) and County health management teams in Busia and Trans-Nzoia Counties. In addition, health facility managers in each facility provided permission to conduct the study. Data privacy was ensured by password protecting the computer.

## Results

### Descriptive analysis

A total of 719 patients were interviewed across the two counties. Of the 719 patients, 11.68% had only diabetes, 65.92% hypertension and 22.39% had both diabetes and hypertension. More than half of the patients were female (75.80%), were informally employed (54.16%), had no form of health insurance (65.23%) and had hypertension (65.92%). Additionally, most patients were between 41 and 60 years (44.37%) and above 60 years of age (47.57%). The mean age was 51.35 years for the diabetes patients, 56.79 years for hypertension and 61.71 years for those with both conditions. Slightly more than a third (33.10%) were subscribed to the National Hospital Insurance Fund (NHIF) – the statutory health insurer (Table 2). There were a total of 1274 outpatient visits with a mean of 1.77 and a median of two visits over a 4-month period.

**TABLE 2:** Patients’ characteristics.

Variable	*n*	% (95% CI)
**Gender**		
Male	174	24.20
Female	545	75.80
**Age category (years)**		
≤ 40	58	8.07
41–60	319	44.37
> 60	342	47.57
**Patient diagnosis**		
Diabetes	84	11.68
Hypertension	474	65.92
Diabetes and hypertension	161	22.39
**Household head education level**		
None	110	15.32
Primary	349	48.61
Secondary	153	21.31
Tertiary	106	14.76
**Employment status**		
Not employed	199	27.79
Informal employment	391	54.61
Formal employment	126	17.60
**Health insurance**		
No insurance	469	65.23
NHIF (National Hospital Insurance Fund)	238	33.10
Private insurance	12	1.67
**Household monthly income in KES**		
999 and below	79	10.99
1000–2999	143	19.89
3000–4999	103	14.33
5000–9999	41	5.70
10 000–19 999	38	5.29
20 000–29 999	15	2.09
30 000–39 999	7	0.97
40 000 and above	7	0.97
Not reported	286	39.78

CI, confidence interval; KES, Kenya Equivalent Shilling.

### Patient costs for seeking outpatient care

#### Diabetes

Overall, the mean annual direct costs (KES 7881 [95% CI: 6950–8812]) for patients with diabetes only were higher than the mean annual indirect costs (KES 683 [95% CI: 425–941]). Under direct costs, medication costs were the highest followed by transport costs (Table 3). In addition, the costs of hiring a paid caregiver were higher (mean annual cost of KES 4600 [95% CI: −4836 to 14 036]) relative to patient productivity losses for seeking care (mean annual cost of KES 508 [95% CI: 399–617]).

**TABLE 3:** Mean diabetes and hypertension annual outpatient costs in Busia and Trans-Nzoia Counties in 2020.

Disease condition	Cost parameter	*n*	Mean			
			Mean (KES)	95 % CI		Mean cost in US$ (2020)
				Lower	Upper	
Diabetes	**Direct medical costs**	84	6493	5635	7352	60
	Out-patient	79	851	639	1063	8
	Medication	78	6130	5321	6940	57
	**Direct non-medical**	79	1353	997	1710	12
	Transport	79	1353	997	1710	12
	**Sub-total (direct costs)**	79	7881	6950	8812	73
	**Indirect costs**	79	683	425	941	6
	Caregiver costs	3	4600	-4836	14 036	42
	Productivity losses	79	508	399	617	5
	**Direct + Indirect costs**	84	8408	7428	9388	78
Hypertension	**Direct medical costs**	474	5944	5347	6540	55
	Out-patient	464	624	536	712	6
	Medication	473	5344	4770	5917	49
	**Direct non-medical**	463	1014	915	1112	9
	Transport	463	1014	915	1112	9
	**Sub-total (direct costs)**	463	6942	6303	7581	64
	**Indirect costs**	463	537	440	633	5
	Caregiver costs	8	2869	-547	6285	26
	Productivity losses	463	487	410	564	4
	**Direct + Indirect costs**	474	7458	6806	8110	69
Diabetes and hypertension	**Direct medical costs**	161	11 264	9446	13 082	104
	Out-patient	158	1169	831	1507	11
	Medication	159	10 244	8524	11 964	95
	**Direct non-medical**	157	1299	1056	1541	12
	Transport	157	1299	1056	1541	12
	**Sub-total (direct costs)**	157	12 539	10 639	14 439	116
	**Indirect costs**	158	631	511	751	6
	Caregiver costs	4	2250	-706	5206	21
	Productivity losses	158	574	481	667	5
	**Direct + Indirect costs**	161	13 149	11 258	15 041	121
Total cost for all patients	**Direct medical costs**	719	7199	6605	7793	66
	Out-patient	701	772	673	872	7
	Medication	710	6528	5961	7094	60
	**Direct non-medical**	699	1116	1022	1 210 281	10
	Transport	699	1116	1022	1210	10
	**Sub-total (direct costs)**	699	8305	7675	8936	77
	**Indirect costs**	700	574	500	649	5
	Caregiver costs	15	3050	1142	4958	28
	Productivity losses	700	509	453	565	5
	**Direct + Indirect costs**	719	8843	8209	9478	82

CI, confidence interval; KES, Kenya Equivalent Shilling.

#### Hypertension

The mean annual outpatient cost for patients with hypertension only per patient was KES 624.00 (95% CI: 534–713) excluding medication. Relative to diabetes costs, the mean annual costs per patient were lower for hypertension. For example, whereas the mean annual medication costs for diabetes was KES 6130.00 (95% CI: 5321–6940), the mean annual costs for hypertension medication were KES 5344.00 (95% CI: 4770–5917). A similar pattern was seen for other cost categories such as direct non-medical costs and productivity losses (Table 3). This suggests that, in general, the mean annual diabetes costs per patient were higher compared to hypertension.

#### Diabetes and hypertension comorbidity

Notably, patients who had both diabetes and hypertension (22.39%) incurred higher direct medical costs compared to patients who had only diabetes or hypertension. For instance, whereas the mean annual costs for outpatient costs per diabetic and hypertensive patient were KES 851.00 (95% CI: 639–1063) and KES 624.00 (95% CI: 536–712), respectively; the mean annual cost for outpatient care for comorbid patients was KES 1169.00 (95% CI: 831–1507) (Table 3). Overall, medication costs were the highest cost driver for patients who had both diabetes and hypertension.

#### Costs by health facility type

At the facility level, both medical and non-medical direct costs for accessing care were highest for private clinics KES 14 235.00 ($131.00) and lowest for dispensaries KES 6390.00 ($59.00) (Table 4).

**TABLE 4:** Mean annual direct cost for accessing care stratified by facility type and disease.

Disease	Cost parameter	Dispensary			Health centre			Hospital			Private clinic		
		*n*	Cost KES	US $	*n*	Cost KES	US $	*n*	Cost KES	US $	*n*	Cost KES	US $
Diabetes	Direct medical	8	4853	45	4	3188	29	60	6564	61	9	8427	78
	Direct non-medical	8	1043	10	4	1575	15	60	1536	14	9	747	7
	Total direct cost	8	5895	54	4	4763	44	60	8100	75	9	9173	85
Hypertension	Direct medical	36	3192	29	28	2935	27	370	5992	55	40	9803	91
	Direct non-medical	36	820	8	28	744	7	368	1026	9	40	1429	13
	Total direct cost	36	4012	37	28	3678	34	368	7019	65	40	11 232	104
Diabetes and hypertension	Direct medical	12	12 804	118	16	11 149	103	114	9902	91	22	19 682	182
	Direct non-medical	12	1050	10	16	1125	10	113	1277	12	22	2082	19
	Total direct cost	12	13 854	128	16	12 274	113	113	11 256	104	22	21 765	201
Total cost for any disease	Direct medical	56	5489	51	48	5694	53	544	6875	63	71	12 690	117
	Direct non-medical	56	901	8	48	940	9	541	1135	10	71	1545	14
	Total direct cost	56	6390	59	48	6634	61	541	8024	74	71	14 235	131

KES, Kenya equivalent shilling.

### Source of prescribed medicines

**FIGURE 1:**
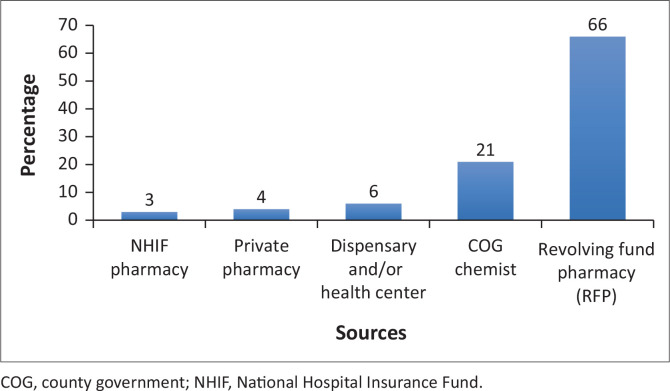
Sources of prescribed medicines.

Patients could obtain drugs from the county government chemist, private chemists nearby or from a community revolving fund pharmacy (RFP) established in the facilities as a complementary drug supply system because of frequent stock outs of drugs in the main chemist and exorbitant cost in the private sector. The RFPs involved a tripartite agreement with a Memorandum of Understanding between the project that provided seed stocks of drugs, the facility that provided a pharmaceutical technologist and the community that conducted advocacy. A *hub and spoke model* was adopted with mini RFPs at lower-level facilities that would be expected to refill drugs from the main RFP when stocks dropped.^36^ Majority of patients (66%) obtained their prescribed medication from the RFP. Further, 21% of patients obtained medication from a county government chemist while only 6% got their medication from either a dispensary or health centre (Figure 1).

### Catastrophic health expenditure

About two in five of the uninsured patients with NCDs incurred CHE annually as a result of the direct costs (direct medical and direct non-medical costs) in outpatient facilities (Table 5). Notably, the incidence of CHE was higher among patients with diabetes and hypertension comorbidity (44.19% [95% CI: 33.90 – 55.00]) compared to diabetes only (35.00% [95% CI: 23.77 – 48.18]) and patients with hypertension only (42.46% [95% CI: 37.17 – 47.93]).

**TABLE 5:** Incidence of Catastrophic Health Expenditure by disease and overall.

Disease Condition	Incidence of CHE				Inequalities in CHE			
	Total observations	*n* incurring CHE	Proportion	[95% CI]	*n*	CIX	[95% CI]	*P*
Diabetes	60	21	35.00	23.77 to 48.18	51	0.066	−0.277 to 0.408	0.708
Hypertension	325	138	42.46	37.17 to 47.93	262	0.081	−0.064 to 0.228	0.271
Diabetes and hypertension	86	38	44.19	33.90 to 55.00	69	0.234	−0.040 to 0.509	0.099

**Overall**	**471**	**197**	**41.83**	**37.43 to 46.35**	**382**	**0.112**	**−0.008 to 0.231**	**0.069**

CI, confidence interval; CHE, catastrophic health expenditure; CIX, concentration index.

### Inequalities in the distribution of catastrophic health expenditure

**FIGURE 2:**
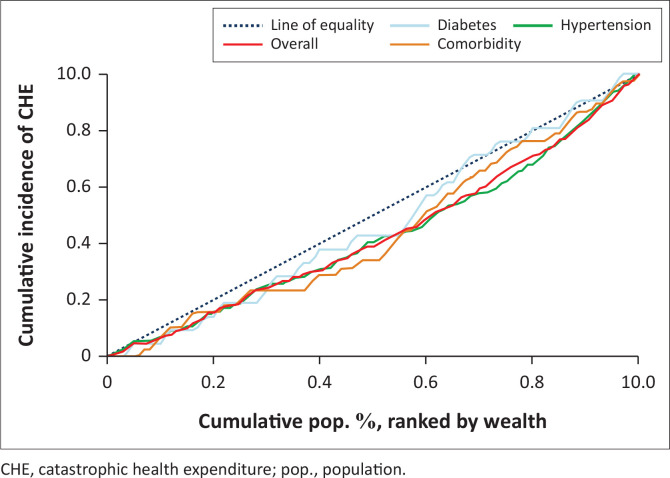
Concentration curves for catastrophic health expenditure by non-communicable disease type and overall.

**FIGURE 3:**
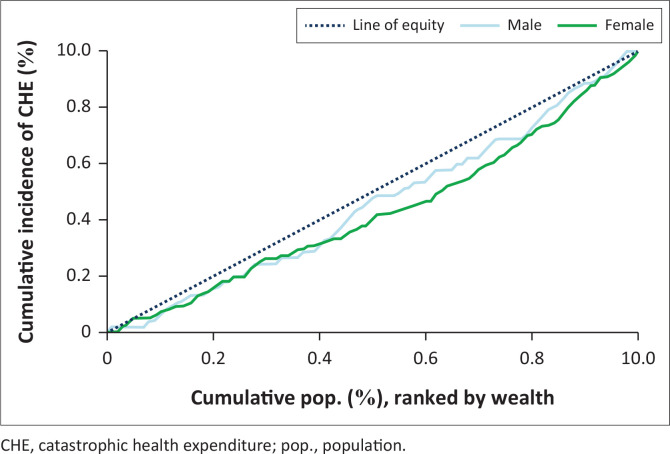
Concentration curves for catastrophic health expenditure by gender.

Overall, the incidence of CHE was more concentrated in the higher wealth quintile (CIX = 0.112 [95% CI: −0.008 to 0.231], *p*-value = 0.069) (Table 5). The highest inequalities were among patients with diabetes and hypertension comorbidity (has the largest absolute concentration index) and its related concentration curve was furthest from the line of equality (Figure 2). Besides, the distribution of the incidence of CHE also varied by gender with women having greater inequalities than men (Figure 3).

## Discussion

The rising burden of NCDs in developing countries and its resultant economic burden has increasingly attracted the attention of key global health actors and policymakers. Specifically, ensuring NCD healthcare services are affordable is critical in addressing the NCDs burden. This study set out to examine the affordability of outpatient costs two NCD conditions across different levels of health facilities. In general, we found that patients with diabetes and hypertension comorbidity incur higher OOP costs particularly in higher level health facilities and private clinics, but all categories of patients experiencing significant CHE. A study by Subramanian et al.^37^ established that screening, diagnosis and treatment costs for NCDs are significantly higher in private facilities relative to public facilities, and that these costs were unaffordable to Kenyans. This may be explained by the subsidies in the public sector through government payments. Despite the cost differences, quality studies do not support the claim that the private sector has better outcomes than the public sector, although it is usually more client orientated and performing better in drug availability.^38,39^

The direct medical costs across the conditions examined in this study were significantly higher compared to the direct non-medical costs. For example, the total mean annual direct medical cost was KES 7199.00 while the total mean annual direct non-medical cost was KES 1116.00. Of note, prescribed medicines costs were the key cost driver under direct medical costs. While this finding reflects what other cost of illness studies for NCDs have established from other African countries,^40,41,42^ this status of affairs is nonetheless worrisome for three reasons. Firstly, it has been established that unaffordability of medicines is the key limitation in the continuity of care for NCDs as patients tend to forego the needed treatment until they have access to funds, resulting in serious medical complications. Secondly, given that more than 20% of the patients in this study obtained their prescribed medication from a government health facility (where the prices of the medicines are subsidised), it is evident that more needs to be done by policy makers to increase budget allocation for healthcare and county managers to ensure lower cost of medication hence increase access and affordability of NCD medicines as medication costs still significantly contribute to financial hardship. For example, the total mean annual medication cost per patient in the overall sample was KES 6528.00 compared to the total mean annual outpatient cost per patient, which was KES 772.00. Further, given that a majority of the patients (66%) sourced their medicines from the RFP, it highlights the inaccessibility of medicines in government facilities because of costs or stock-outs and reinforces the utility of RFP in improving access to NCD medicines especially in rural areas.^36^ Finally, the high medication costs observed in this study is a concern given that previous studies have established the existence of a wealth gradient in the access to medicines for NCDs in developing countries. A study conducted in eight counties in Kenya, for example, found that poorest households, relative to wealthier households are more likely to have limited access to medication and had to pay higher costs overall to obtain medication for hypertension, diabetes and asthma.^43^ Moreover, in as much as the finding of this study suggested that most patients obtained their prescribed medication from a government health facility and the RFP, another study conducted in Kenya showed that NCDs medicines were more readily available in the private sector compared to public health facilities.^44^

Transport costs were also another cost driver in this study, highlighting the need to have NCD services closer to the patients. Indeed, transport costs were higher than outpatient costs for NCDs like diabetes and hypertension. Whereas the mean annual outpatient cost was KES 772.00, the mean annual transport cost per patient was KES 1116.00. Previous studies have established that transport cost increases the proportion of NCD patients incurring CHE in Kenya, and transport-related expenses are therefore a likely barrier in seeking healthcare for NCDs.^14,15,45^ Another study that evaluated whether health financing reforms that target the poor benefit them identified transport cost as one of the key barriers hindering the poor benefiting from these reforms.^46^ It is worthy to note that although the indirect costs either because of seeking healthcare or hiring caregivers because of the NCDs are lower than the direct costs in this study, they also contributed to the economic burden experienced by households. Elsewhere, a South African study has shown that productivity losses because of long waiting times at health facilities are likely to reduce the demand for NCD healthcare services.^47^

While hypertension and diabetes outpatient services are presently majorly offered in public secondary care facilities and private clinics, our findings suggest that these services as currently distributed are not affordable to most patients, especially those with comorbidity. Overall, 41.83% of the uninsured patients used more than 10% of their income in meeting healthcare costs. Elsewhere, it has been shown that Kenyan households with a member who has a NCD are twice as likely to incur CHE compared to households without a member with a NCD or chronic ailment.^45^ A study comparing CHE by disease area in six countries showed that the share of CHE from NCDs rises with the share of DALYs, defined as years of life lost to disability and death. Non-communicable disease CHE was also more likely to arise from many visits rather than a one-time event, when compared to communicable diseases.^48^ Given the high levels of poverty in Kenya and the low prepayment mechanisms in place to cushion patients from financial hardship because of healthcare payments (80% of Kenyans are not subscribed to any health insurance scheme),^49^ county and national governments need to put strategies to improve the population’s financial risk protection in accessing NCD services. For example, given that most people reside in rural areas in Kenya, dispensaries and health centres should be the focus in increasing service delivery for NCDs as we have shown here that both the direct and indirect costs of care at these primary level facilities are lower than the secondary level of care.

It is noteworthy that this study was undertaken in facilities that had substantial investment in the care cascade for hypertension and diabetes through the PIC4C project. As a result, the cost of care may be lower than that in many other facilities as drugs were made available through the community revolving pharmacies at a lower cost than in private yet in most public settings stock outs are frequent with most patients accessing NCD medication in private. As a result, it is possible that the cost of care is even higher in the rest of the counties.

This study had several limitations. Firstly, as the respondents were recruited in facility chronic care clinics, patients not on care and those that could not afford these care services may be underrepresented. Secondly, because of the recall periods, results may be affected by recall bias. Thirdly, the reliance on an official minimum wage to calculate productivity losses for those without a stated income could have overestimated indirect costs. Lastly, as some respondents did not have income estimates, the CHE estimates should be interpreted with caution. Nonetheless, the data presented are potentially useful inputs in economic evaluation models that require patient costs.

## Conclusion

Patients with NCDs still experience substantial costs when accessing healthcare services in Kenya. Evidence from the two Western Kenya counties indicates that NCD comorbid patients incur substantial OOP costs in seeking healthcare, with the poor households spending a higher proportion of their income in receiving health services for NCDs. Measures to ensure affordability of outpatient NCD services should be urgently implemented while concerting prevention and screening efforts to reduce NCDs burden in the country. Provision of NCD services at PHC facilities could ensure achievement of both. Estimates from our study can be used to inform the inclusion of NCD services in the rollout of the UHC scheme in Kenya.
